# Specimen Design and Characterization for Thin-Walled Components in Very-High-Cycle Fatigue Regime: Aluminium 6082 Case Study

**DOI:** 10.3390/ma19020273

**Published:** 2026-01-09

**Authors:** Felipe Klein Fiorentin, Rita Dantas, Jorge Wolfs Gil, Aida Beatriz Moreira, Francisco Matos, Andrea Piga Carboni, Thiago Antonio Fiorentin, Abílio Manuel Pinho de Jesus

**Affiliations:** 1 Department of Mobility Engineering, UFSC (Federal University of Santa Catarina), Joinville 89219-600, Brazil; 2 Institute of Science and Innovation in Mechanical and Industrial Engineering, Campus da FEUP, Dr. Roberto Frias Street, 4200-465 Porto, Portugal; 3 Faculty of Engineering, University of Porto, Dr. Roberto Frias Street, 4200-465 Porto, Portugal; 4 Institute for Sustainable Construct, 4200-465 Porto, Portugal

**Keywords:** VHCF, fatigue, aluminium alloy, 6082, thin-walled components, gigacycle

## Abstract

Rapid characterization of high-cycle fatigue behaviour is of great interest, since conventional methods for developing S-N curves for longer fatigue lives are both costly in time and financial resources. Ultrasonic fatigue testing offers a promising alternative by enabling S-N curve evaluation in a fraction of the time, often hundreds of times faster, due to its high testing frequencies. Nevertheless, this technique presents specific challenges, including material overheating and limitations in specimens’ geometry. Most ultrasonic fatigue studies employ hourglass specimens; however, this geometry restricts the testing of sheets and thin-walled components, which are increasingly used for their reduced mass and high stiffness-to-mass ratio. To overcome this limitation, the present work introduces a methodology for designing and testing flat specimens and corresponding gripping systems tailored to such components. The procedure is demonstrated for an aluminium alloy (6082), and preliminary experimental fatigue results are presented and compared with literature.

## 1. Introduction

Components subjected to variable loads present a challenge for engineering design and prediction because once these loading conditions lead to a damaging effect in these parts the materials fail due to fatigue. Fatigue characterization is fundamental in engineering because many critical components (such as aircraft wings, rotating shafts, bridges, and biomedical implants) fail due to cyclic loading rather than single overloads (monotonic loads) [1]. Under repeated stress oscillations, microscopic cracks nucleate and grow until catastrophic fracture occurs [2], often without prior macroscopic warning. The ability to experimentally characterize this behaviour, evaluate microstructural influences, and predict fatigue life is essential for preventing sudden failure, ensuring safety, and optimizing maintenance strategies [3]. The accurate fatigue life assessment not only reduces the risk of unexpected accidents but also avoids over-conservative designs that increase cost and weight [4].

By the end of the 20th century, attention increasingly turned to the very-high-cycle fatigue (VHCF) regime, which typically involves fatigue lives beyond 10^7^ cycles [5]. It was observed that the so-called fatigue limit of some materials (like steels) was mostly a convention, mainly observed due to the limitations of the excitation frequencies of the machines (which would take hundreds of years to perform tests in the range of 10^9^ cycles, for example) [6]. Many modern components, such as high-speed bearings, aerospace engine parts, ultrasonic actuators, and thin-walled structures (like rotor blades, for example), experience service lives in this regime [7]. Unlike conventional high-cycle fatigue, VHCF is characterized by different crack initiation mechanisms, often shifting from surface defects to internal inclusions (depending on material properties), with distinctive fracture features such as fish-eye regions and fine-granular areas [8]. Factors such as inclusion size, loading frequency, temperature, and environment strongly affect VHCF behaviour, making extrapolations from conventional S-N data unreliable [9]. Consequently, VHCF characterization is indispensable for ensuring structural integrity in long-life applications [10]. In order to achieve such a high number of cycles, it is essential to apply high excitation frequencies due to the total characterization time required [11]. To achieve such high frequencies, ultrasonic actuators like piezoelectric crystal technology are often used.

The ultrasonic fatigue testing involves some challenges, like the need to design specimens for a specific frequency [12]. Additionally, no external force is applied to the specimen; it is loaded by its own inertial forces, so the specimen’s own inertia directly impacts its stress field. Due to the high excitation frequencies, ultrasonic fatigue specimens may also experience high temperatures during tests [13], and high temperature gradients [14], which will lead to changes in the fatigue behaviour of materials [15].

A standard design choice for ultrasonic fatigue testing is hourglass specimens, where analytical solutions regarding natural frequencies and stress distribution are available [16]. However, some part geometries do not allow the manufacturing of an hourglass specimen. This is a case for thin-walled specimens, due to their low thickness. Additionally, this small dimension also creates challenges regarding the specimen’s attachment to the machine (horn) [17], where it is not possible to manufacture threads in the specimen (due to its low thickness). For these kinds of complex specimen geometries, no analytical solution is available. Consequently, designing specimens for testing thin-walled components might be challenging. Some authors have proposed adaptations to the fatigue machine, like adding a second horn [18]. Others have attempted to use typical ultrasonic testing by soldering or using adhesives in order to attach the specimen to the rest of the ultrasonic system [19]. This solution can be very time-consuming and more prone to error, since the soldering (or bonding) process must be performed in every specimen. Other authors have proposed an intermediary component (gripper), responsible for the connection between the specimen and the ultrasonic fatigue system (like the horn or booster) [20].

Regarding the gripping specimen proposed by Tofique et al. [20], one of its main advantages is that the relation of the applied displacement (machine load) and the stress level at the testing section can be controlled by changing the gripper material and/or geometry. Moreover, the contact forces can be controlled by the interference level and bolt pre-load. When analyzing the proposed design limitations, it required relatively tight tolerances (mainly due to the assembly required between the specimen and the grippers). Moreover, the specimen and gripper are held together only by frictional forces (due to the normal load resulting from bolt pre-load and interference); consequently, if this force is not large enough, relative motion will happen between the components, resulting in severe variation in stress levels at the testing section and variations in the system’s natural frequency. This problem is not present in solutions where the specimen and the fixturing system are held together by other joint methods, like soldering/brazing or adhesion bonding.

Thin-walled components are widely employed in automotive, aerospace, and marine systems, where weight reduction and high stiffness are critical aspects [21]. By reducing wall thickness, engineers can achieve lighter structures and higher material efficiency [22]. However, this geometry inherently increases susceptibility to local buckling, stress concentrations, and fatigue crack initiation under cyclic loading [23]. Experimental and numerical investigations showed that thin-walled structures, such as beams, panels, and tubular elements, often require distinct fatigue analysis approaches (when compared to bulk materials) [24].

Among lightweight structural materials largely used in thin-walled applications, Aluminium 6082 alloy (Al-Si-Mg series) is particularly relevant due to its excellent strength-to-weight ratio [25], corrosion resistance, and weldability, which make it a standard choice in transport, marine, and offshore structures [26]. Its widespread use in extruded thin-walled sections for bridges, railway cars, and automotive components highlights the need to evaluate its fatigue performance [27], under both high-cycle and very-high-cycle regimes. Consequently, understanding its fatigue response is indispensable for safe lightweight design in industries demanding high durability under cyclic service conditions [28]. Interesting studies regarding the VHCF characterization of the 6082 alloy for hourglass specimens (standard solution) were performed by Dimitrov et al. [29] and Peliteiro [30].

Therefore, a methodology was developed to overcome the lack of information regarding the procedure to design specimens from thin-walled components, which could be considered to characterize the behaviour/performance of a material in the VHCF regime. The effect of main material properties as well as geometry was assessed and considered throughout the development of this procedure.

The main goal of the present work is to develop a comprehensive design methodology for testing thin-walled components in the ultrasonic fatigue regime. The impact of key factors, such as material properties and thin sheet specimen main dimensions, on the resonance frequency and stress amplitudes will be discussed. Additionally, the proposed design methodology will be applied to a real case scenario, the VHCF characterization of an aluminium alloy, 6082, where specimens were obtained from a thin sheet. This will be performed to validate the proposed methodology, as well as the specimen design and testing conditions. It is worth noting that although the specimens analyzed were obtained from a thin-rolled sheet, the same design and fatigue testing methodology can be extended to the characterization of other thin-walled components produced by different manufacturing processes, such as casting or additive manufacturing.

## 2. Materials and Methods

This section will provide a detailed discussion regarding the important parameters that should be analyzed during the design process for thin-walled VHCF specimens. The relevance of geometric and material parameters for the specimens and gripping system will be discussed. Next, details regarding the manufacturing of the specimen and fixturing system will be provided. Lastly, the manufacturing process and experimental process will be explained. The experimental procedure will provide details about both the VHCF behaviour of the material and the validation of the numerical model as well.

### 2.1. Base Alloy Description

The material investigated in this work is an EN AW-6082 aluminium alloy (EN AW-Al Si1MgMn), classified according to EN 573-3:2019 [31]. The alloy was supplied as a rolled sheet in a peak-aged condition, nominally equivalent to a T6/T5x temper, commonly used in structural applications requiring a good balance between strength and ductility. The nominal chemical composition of the EN AW-6082 alloy, as specified by the supplier and consistent with EN 573-3:2019, is given in Table 1.

The microstructure of the base alloy was characterized on specimens extracted from the rolled EN AW-6082 sheet. Small specimens were cut from the top surface of the fatigue specimens, in the clamping area and away from regions affected by crack initiation and growth, as well as from the longitudinal section of the sheet. The samples were metallographically prepared using a standard grinding and polishing procedure and subsequently chemically etched with Weck’s reagent to reveal the α-Al grain structure and the distribution of second-phase particles.

Microstructural examinations were carried out using a scanning electron microscope (FEI QUANTA 400 FEG (FEI Company, Hillsboro, OR, USA)) operated in secondary electron (SE) and backscattered electron (BSE) modes at an accelerating voltage of 15 kV. BSE imaging was employed to enhance atomic number contrast and to reveal the distribution of intermetallic particles and defects in the Al–Mg–Si α-Al matrix. Selected areas were further analyzed by energy-dispersive X-ray spectroscopy (EDS) to identify the chemical composition of the coarse intermetallic particles and to distinguish Fe/Mn-rich phases from Mg–Si-rich particles.

The hardness of the base material was assessed by Vickers microhardness testing according to EN ISO 6507-1:2023 [32], using a load of 1 kgf (HV 1). Seven indentations were performed on the top surface of the fatigue specimen, in the clamping area and in regions not affected by fatigue damage, with a spacing of at least three times the diagonal length between adjacent indents. The average hardness and standard deviation were calculated from these measurements and used as reference values for the peak-aged condition of the EN AW-6082 alloy.

It is important to highlight that, since the main work is focused on a methodology for designing and testing specimens for the VHCF regime, the material could provide preliminary information. Once the 6082 is an aluminium alloy, it is well-established that no fatigue limit will be present during the fatigue tests. Instead, a smaller slope is expected at the S-N curve for a higher number of cycles.

### 2.2. Specimen Design

**Figure 1:**
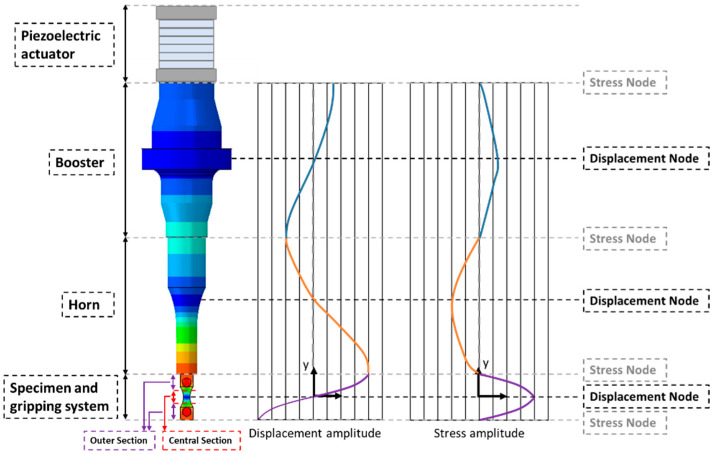
Ultrasonic fatigue machine’s main parts and the respective displacement and stress distribution along length.

The specimen’s design must take into account several factors, like machine capabilities, testing material properties, and manufacturing and fatigue tests’ particularities. Figure 1 presents a typical schematic of the main components of an ultrasonic fatigue test. One of the particularities of ultrasonic fatigue testing is related to the specimen’s design, as it needs to have one of its natural frequencies equal to the machine excitation frequency. These systems usually consist of the following:

- An actuator, which is usually piezoelectric due to the high excitation frequencies required, is responsible for providing the displacement excitation.
- A booster and a horn: These components are typically responsible for amplifying the displacement amplitude generated by the actuator. In some machines, these two components can be combined into one. Additionally, the booster also holds the attachment region (fixation) between this dynamic system and the rest of the machine.
- A specimen: This component should be designed to have one of its natural frequencies within the machine excitation frequency range. Due to this constraint, some challenges are associated with the machine design. Additionally, levels of stress amplitudes present in the specimen must be large enough to induce fatigue crack initiation and consequently generate a S-N curve. Lastly, the specimen must be rigidly attached to the horn of the machine. This connection is usually made using a thread (usually at the specimen) and a threaded hole (commonly present at the horn). For the present case, due to the low thickness of the thin-walled specimen, it is not possible to machine threads at the specimen, so an intermediate gripping system for the specimen was used as a fixturing solution (although the gripping system has other functions, which will be discussed later).

Figure 1 also presents displacement and stress amplitudes along the machine’s length (the colormap refers to the displacement magnitude). It is important to notice that near null stress amplitudes are present between the component connections (like the booster and horn, or horn and specimen contact region). Lastly, it can be seen that the maximum stress amplitudes will occur at the specimen’s middle length, and, therefore, this is the region where crack nucleation and propagation tend to occur.

Hence, it is fundamental to take into account that the specimen must be designed to have one of its natural frequencies coincident with the excitation frequency (the natural frequency of the entire system). For a one-degree-of-freedom system, the natural angular frequency can be written as follows:

(1) $$\omega_{n}=\sqrt{\frac{K}{M}},$$

where *K* is the stiffness, and *M* is the mass. This means that, in order to increase the natural frequency of a system, the stiffness must be increased or the mass reduced. Regarding the influence of material properties, the stiffness is directly proportional to the elasticity modulus of the material, while the mass is proportional to the material density. For several degrees of freedom, an analogous approach can be used. Regarding the stiffness, for a bar element, it can be written as follows:

(2) $$K=\frac{EA}{L},$$

where *E* is the elasticity modulus, *A* is the cross-sectional area, and *L* is the length. This analysis can also be used for a specimen that is being loaded in a direction parallel to its longitudinal dimension. Consequently, if the goal is to increase the specimen stiffness, the design approach will consist of increasing the cross-sectional area or reducing its length.

For an undamped, single-degree-of-freedom system, the differential equation of motion can be written as follows:

(3) $$M\ddot{x}+Kx=F_{ext},$$

where x and $\ddot{x}$ are the displacement and its second derivative with respect to time (acceleration), respectively, and *F_ext_* is the external force. Analyzing Figure 1, it can be seen that the central section of the specimen presents the smallest displacement amplitudes. Therefore, at this central portion of the specimen, the inertia term ($M\ddot{x}$) of Equation (3) is very small, being that the second term (Kx) is much more prominent. The opposite is true for the outer region of the specimen, where the influence of the inertial term is more noticeable than the stiffness term. This analysis can provide an initial insight into the influence of each region and parameters on the final design and behaviour of the specimen.

**Figure 2:**
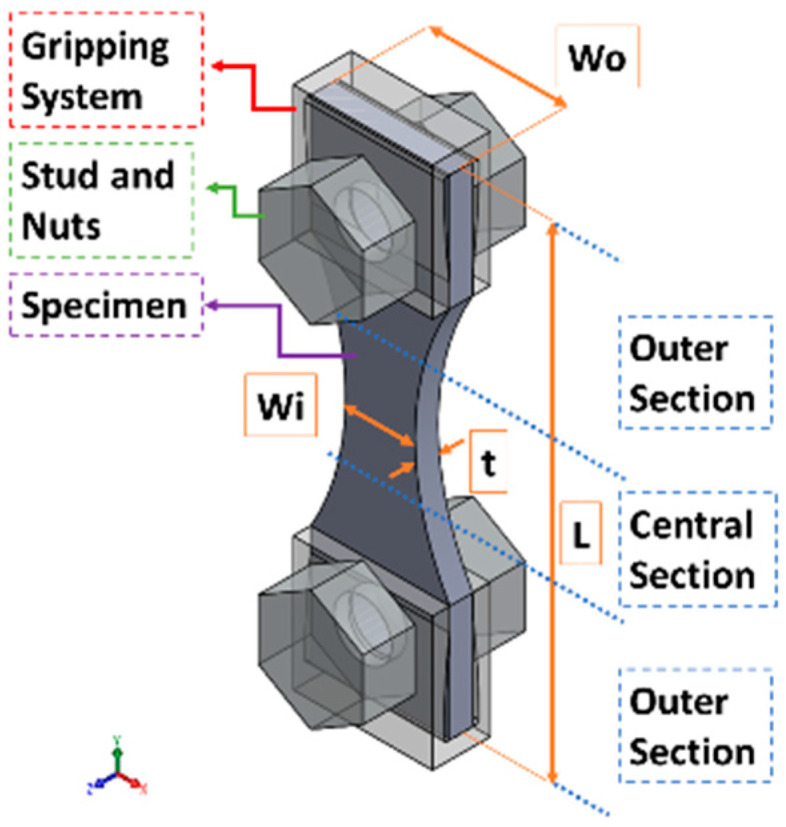
Specimen and gripping system main parts and dimensions.

Regarding the testing of flat specimens in ultrasonic fatigue, one of the main concerns is associated with attaching the specimen to the rest of the machine (the threaded connection presented in the horn, for example). This problem can be solved by the “horse-shoe” fixturing (based on the design proposed by Tofique [20]), which is portrayed in Figure 2. It is composed of four main components: a specimen, two gripping parts, a pair of studs, and two pairs of nuts. In order to maintain the specimen symmetry in the three main Cartesian planes, using a pair of bolts and nuts was not possible (due to differences in geometry and mass of the bolt head and the nut, the specimen would lose its symmetry along one of the main planes). For the gripping system, the studs and nuts were applied to the upper and lower parts of the specimen to ensure symmetry in the three main Cartesian planes, even if the lower part was not used to attach to anything. Figure 2 also presents the main dimensions of the specimen, with *L* being the specimen length, *t* its thickness, *W_i_* its testing section width, and *W_o_* its gripping region width. For the present analysis, the gripping section width used was the same as the specimen’s at this region (although a different width could be used without any major concerns). All these dimensions impact the specimen’s final natural frequencies and the stress amplitudes.

**Figure 3:**
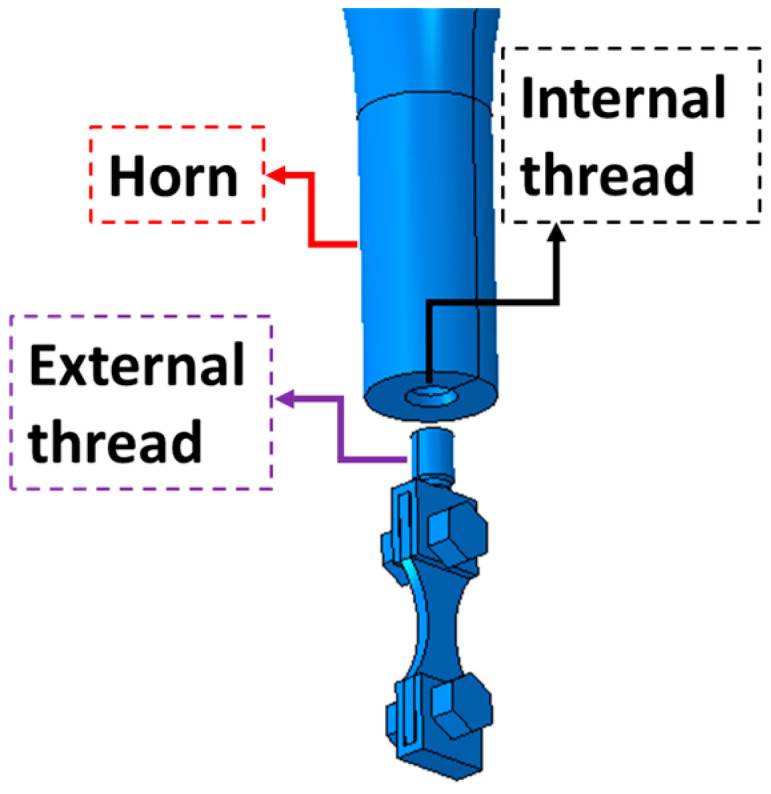
Fixation between specimen gripper and ultrasonic machine horn.

The gripping parts also need to be different, since the one in contact with the horn must have a threaded connection. Figure 3 presents an overview of the connection between the horn and the top gripping system. As can be seen in Figure 1, this thread connection is located at a region of very low stress amplitude, and therefore, this connection is not critical from a stress point of view.

**Figure 4:**
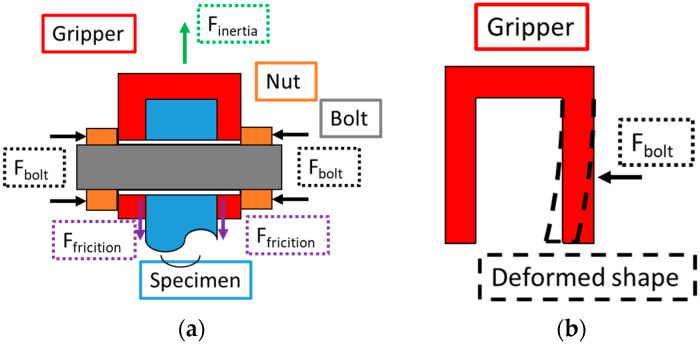
Normal and friction forces at the gripping system: (**a**) Main components and forces; and (**b**) schematic view of gripping system bending diagram.

Regarding the interaction between the specimen and gripper, Figure 4 presents a schematic view of the forces involved and how they can be modelled. For the present case, no contact between the bolt and the gripper and between the bolt and the specimen was present (through hole). Therefore, these two components are held together by the friction force between the two pairs of surfaces (specimen front and a gripper internal face, and specimen back and the other gripper internal face), as shown in Figure 4a. The friction force can be expressed as follows:

(4) $$F_{friction}=\mu \;N,$$

where μ is the friction coefficient, and N is the normal force. The normal force is generated by the bolt pre-load (if no interference was presented between the gripper and the specimen prior to bolt load). Additionally, if a gap is present between the gripper and the specimen (to facilitate the assembly process, for example), a portion of the bolt pre-load force will be required to deform the gripper and ensure the contact between it and the specimen, as shown in Figure 4b. This will lead to a smaller resulting normal force, consequently reducing the friction forces.

Additionally, due to the relative position of the gripper according to the specimen, with the first being located farther away from the specimen centre, leading to higher displacement and acceleration amplitudes (Figure 1), a longitudinal force will appear due to the gripper’s inertia and acceleration. This force will have a tendency to generate relative motion between these components; therefore, it must be counteracted by the friction force. Here, for simplicity, this force will be referred to simply as F_inertia_, or the longitudinal separation force. If the longitudinal separation forces between the specimen and the gripper are smaller than the friction forces, the gripper and specimens will remain with no relative motion between them.

A brief analysis of the main parameters’ influence on the specimen’s behaviour will be presented here, which may act as a design guide for analogous solutions:

- Specimen elasticity modulus: As observed in Equations (1) and (2), a higher elasticity modulus will increase the specimen stiffness and, consequently, the natural frequency. Since the final goal of the ultrasonic fatigue testing is to characterize a given material, this parameter cannot be changed. Regarding the impact of stress, since the elasticity modulus will increase the specimen stiffness (less displacement associated with a given load), it will also increase the stress for a certain displacement amplitude (higher specimen stress gain). Looking at Equation (3) and the following discussion, once the elasticity modulus only impacts the stiffness term, these material parameters will be very influential in the central portion of the specimen (where stiffness plays a key role) and will have little impact on the outer regions.
- Specimen density: The density will have an opposite impact on the natural frequency when compared with the elasticity modulus; higher material densities will increase the total mass and, according to Equation (1), will reduce the natural frequency. Due to being directly associated with the mass, and following the discussion regarding Equation (3), the density will have a higher impact on the outer region. This information will be relevant for the following discussion regarding the impact of the fixturing system.
- Specimen length (L): As observed in Equation (2), a longer specimen will decrease its stiffness and, consequently, reduce its natural frequency. Moreover, since increasing the specimen length will reduce its stiffness, the maximum stresses in the specimen will be reduced (lower specimen stress gain).
- Specimen testing section width (W_i_): The impact of reducing the testing section width will decrease the natural frequency (lower stiffness) and increase the overall stress (higher stress gain).
- Specimen gripping section width (W_o_): Since it is located at the outer region, increasing the gripping section width will increase the overall mass (especially if the width of the gripping system is also increased), reducing the natural frequency. Due to the growth in the overall mass, the stress gain will also be increased (larger inertia forces).
- Specimen thickness (t): The thickness will increase both the specimen stiffness and mass. If no gripping system was present, the thickness would have no impact on the specimen’s natural frequency (once, it would impact the mass and longitudinal stiffness equally). For the present case, there is also a gripping system present, making the analysis more complex; increasing the specimen thickness will also increase the gripping system’s overall size (and mass), and the impact must be analyzed case by case. In this study, the specimen will be obtained from a sheet, and consequently, the thickness value used will be fixed. It is important to emphasize that a specimen with low thickness might be difficult to perform tests on in machine setups, like the present one (ultrasonic fatigue with one horn), since the specimen will have very low lateral stiffness (resulting in several bending modes).
- Studs, nuts, and gripping system dimensions: Increasing the overall dimensions of these three components will increase the total mass, which will reduce the natural frequency. Since these components are located at the outer region of the specimen (as shown in Figure 1), this increase in mass will have a strong impact on loading (according to Equation (3), due to the inertia term) of the specimen, increasing the maximum stress (higher stress gain). Additionally, higher mass at the gripping region would also lead to greater longitudinal separation forces between the specimen and gripper (due to the higher gripper inertia). It is important to emphasize that using a bolt and nut with higher diameters will increase the maximum pre-load, leading to higher friction forces between the gripper and specimen. This is a desired characteristic for the test. Additionally, this normal force can be enhanced even further by utilizing higher resistance classes for the bolt and nuts.
- Stud, nut, and gripping system density: The impact of using a denser material will be similar to increasing these components’ dimensions (previously discussed), leading to a higher total mass (and lower natural frequency, consequently).

Table 2 presents a summary of the influence of the main material parameters and dimensions in the specimen’s stiffness, mass, and natural frequency (regarding the mode of interest).

With all information regarding the impact of each dimension and material property (for the specimen and gripper) on the specimen’s natural frequency and stress amplitudes, it is now possible to advance with some design specifications. In order to maximize normal forces, at the contact, high-class bolts (12.9) and nuts (12) were used. This allows the increase in the normal force without the need for increasing the gripping system mass (which would lead to greater longitudinal separation forces between the specimen and gripper). Regarding the nominal diameter, an M5 bolt was used. If higher friction forces are required, increasing the bolt diameter would greatly improve the normal forces (due to the higher pre-load), although the total gripping mass would also be increased (requiring an iterative process to adjust specimen natural frequency).

In order to reduce the gripping system’s total mass (and consequently the maximum stress amplitudes at the specimen), a titanium alloy (Ti4AlV6) was used for the grippers. Once the elasticity modulus of titanium is approximately half that of the one for steel, the bending stiffness of the gripping system would also be reduced, as shown in Figure 4b, which is also a desired effect.

As previously discussed, minimizing the gripping system’s total mass is often a desirable effect, as it reduces the longitudinal separation forces between the specimen and gripping system. However, this lower overall mass will lead to lower forces at the testing section of the specimen. Consequently, it might be necessary to reduce the cross-sectional area at this region in order to increase the stress amplitudes. Once the specimen thickness is fixed for the present analysis, this can be achieved by reducing the testing section width, *Wi*.

Machine operating conditions and mechanical properties of the specimen material are fundamental to developing a design and fatigue testing procedure. Table 3 presents a summary of these properties. The fatigue tests will be performed on an aluminium alloy, 6082. As previously discussed, its density and elasticity modulus impact both the natural frequencies and stress amplitudes. The ultimate tensile strength (UTS) will be used as a guide to the desired stress levels, which will not be required to be higher than this value. As previously discussed, the 1st longitudinal mode of vibration of the specimen and gripper set must be within the machine operating frequency. Subsequently, it is important to take into account the maximum and minimum displacement amplitudes that the machine is capable of achieving. This is essential once it is directly linked to the maximum and minimum stress amplitudes for a given specimen geometry. The relation between the resulting stress and the applied displacement amplitude is often called specimen gain, and it is a constant value that can be estimated by numerical analysis.

The design process for VHCF is usually an iterative process. First, the specimen geometry must be adjusted to have the desired natural frequency (for the present case, the 1st longitudinal frequency) in resonance with the machine excitation frequency. Next, whether the excitation condition (minimum and maximum machine excitation displacement amplitudes) is within the specimen’s desired test range (stress) must be determined. As previously detailed, this relation is often summarized as the specimen gain (the relation between stress amplitudes for a given displacement). If the obtained stress levels are inferior to the ones necessary for fatigue tests, the specimen must be redesigned. The same happens if the minimum stress obtained is higher than the desired stress amplitudes for fatigue tests.

Regarding the thin-walled specimens and the applied gripper designs, the friction forces between the specimen and gripper must also be analyzed to determine if they are large enough to avoid any relative motion between these two components. If the friction forces are not large enough, a new design is required. In order to maximize the friction forces, some solutions can be applied, such as the following:

- Maximizing the friction force between the surfaces: This can be achieved by increasing the friction coefficient between them, or increasing the resulting normal force, as shown in Equation (4). The normal force can be improved by utilizing a larger bolt pre-load (larger bolt diameter or higher-class bolt) and/or reducing the gripper stiffness and gap between the gripper and the specimen. Moreover, the specimen can be assembled using interference by using a specimen thickness that is larger than the gripper gap, assuring an additional contact force.
- Minimizing the longitudinal separation force between the specimen and gripper: In order to accomplish this, the total inertia of the gripping system (gripper, bolt, and nuts) can be reduced. It is important to emphasize that this mass reduction will also affect the system’s resulting natural frequency (increasing it, due to mass reductions in the system, without any significant changes in mass), and the stress at the testing section (which will be reduced, due to the lower inertial term).

Lastly, after designing the specimen for a given natural frequency and gain (relation between resulting stress and applied displacement), it is necessary to verify if there are no other natural frequencies close to the one of interest. If other frequencies are very close to the machine working frequency, it means that the specimen might not be excited at the desired natural mode and frequency, or that its resulting vibration movement will be a combination of both the desired and undesired modes. If that is observed (other frequencies close to the desired specimen natural frequency), a specimen redesign will be necessary.

### 2.3. Numerical Model

**Figure 5:**
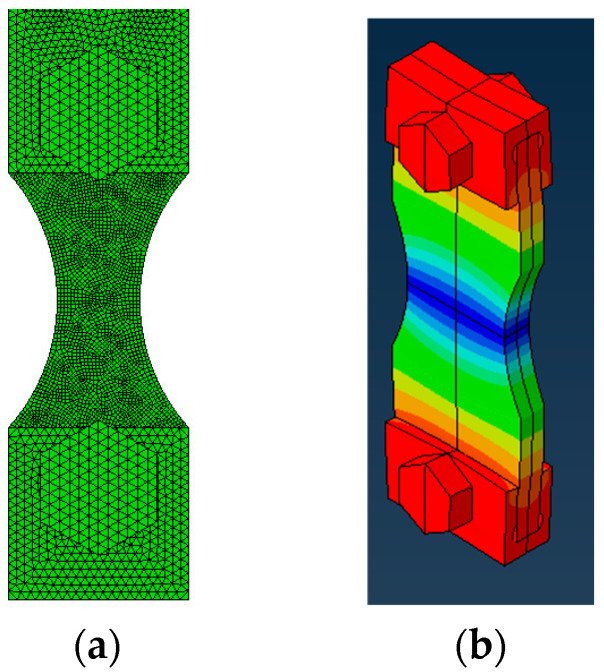
Symmetry model: (**a**) mesh overview; and (**b**) 1/8th symmetry.

The Finite Element Method (FEM) was used to assess the natural frequencies, where a modal analysis was performed, and the displacement/strain/stress fields for each specimen and gripping system design were obtained. Three-dimensional linear 4 and 8 nodes (C3D4 and C3D8 elements of Abaqus 2023 software, respectively) were used, and approximated element sizes of 0.20 mm were considered to mesh the specimen (after a mesh convergence analysis), shown in Figure 5a, while the bolts and gripper system used linear 4 nodes (C3D4) and an element size of 0.40 mm. Later, the mesh convergence analysis will be discussed with the mode details.

The iterative design consisted of designing a preliminary geometry and calculating its natural frequency. Afterwards, the specimen length was adjusted in order to reach the desired longitudinal natural frequency (around 20 kHz). When, at the present step, the only mode of interest was the 1st longitudinal mode, it was possible to use symmetry at the three main orthogonal planes (front, top, and side), as shown in Figure 5b. In addition to the symmetry around the 3 main orthogonal planes, no other boundary condition (restrictions) was applied. Moreover, only the specimen and gripping system were modelled at this step. A tied contact was used between all pairs of contacts (specimen and gripper, and gripper and bolts). Next, the relation between the specimen stress and displacement (specimen gain) was computed. Figure 5b also presents an example of a longitudinal displacement magnitude field, where it can be seen that the central section of the specimen presents the smallest displacement, while maximum displacement was obtained at the specimen’s top and bottom ends (for the 1st longitudinal vibration mode).

After analyzing the natural frequency and specimen gain, it was necessary to ensure that the friction force between the specimen and gripper system was enough to hold them in place. Once the bolt pre-load is known, it is possible to compute the normal forces at the specimen. Consequently, with the friction coefficient, it is possible to calculate the friction force. The longitudinal contact forces between the specimen and gripper were obtained from numerical simulation. This longitudinal contact force was then compared with the friction force. If the friction force found is higher, it can hold the specimen and gripper together. If not, it is necessary to redesign the specimen and gripper set. As discussed before, this new design could improve the normal forces (like increasing the bolt pre-load, for example) or reduce the longitudinal contact forces (for example, reducing the inertia of the gripping system).

If this analysis is successful, it means that the specimen is within the operating frequency range of the machine, and by using the gain, it is possible to compute the minimum and maximum stress amplitudes that could possibly be achieved during tests, and no sliding between the specimen and gripper would be present. If this stress range is within the desirable range for S-N tests, this step is finished (for that specimen geometry).

**Figure 6:**
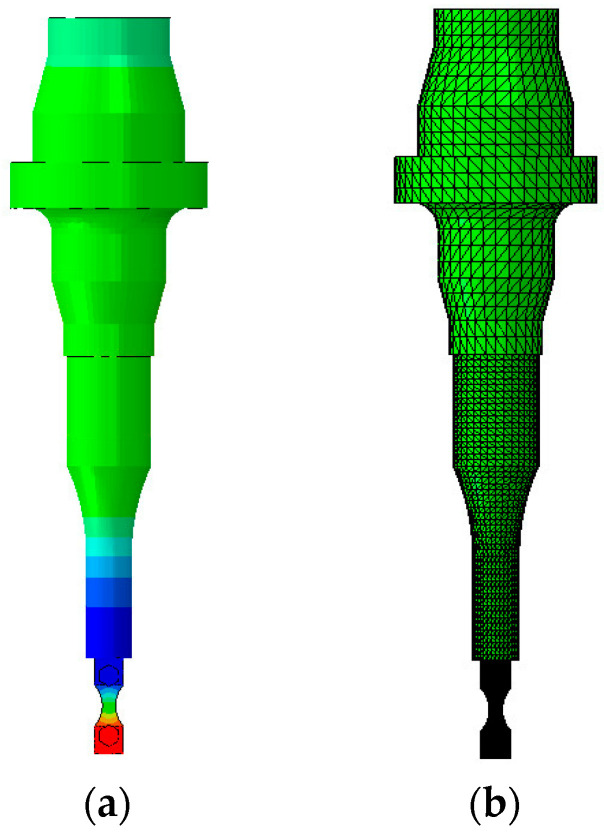
Full model: (**a**) Overview; and (**b**) mesh overview.

The next step consists of a more complete simulation, without any symmetry (full model), and encompasses two machine elements, the booster and horn (as shown in Figure 1, for example). Figure 6 presents an overview of the full model, where the mesh is presented in Figure 6a, while Figure 6b shows an example of the longitudinal displacement amplitudes. The specimen and gripping system mesh size was the same as in the symmetry model, while the horn (component attached to the gripping system) element size was 1.50 mm, and the booster’s size was 3.00 mm (component at the top portion of Figure 6). This simulation was performed without any restriction (free-condition), which is a situation very similar to what the machine system experiences during tests (where the dynamic components are attached to the rest of the machine at a node region, in order to minimize the fixturing system’s influence). This analysis can provide more reliable calculations regarding the natural frequency and displacement/strain/stress fields. However, the main goal of this analysis is to verify if there are any vibration modes with their corresponding natural frequencies close to one of the modes of interest (approximately 20 kHz). If a mode close to the one of interest is found, it means that the specimen might experience stress fields (due to the different vibration modes) different from those desired.

**Figure 7:**
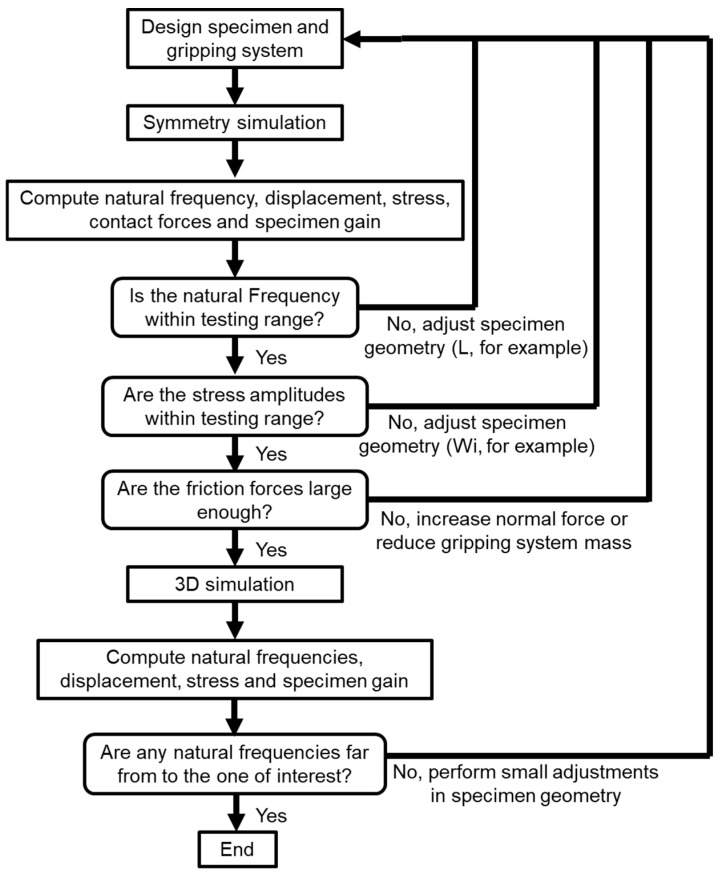
Specimen design flowchart.

Additionally, uncertainties related to the material properties or manufacturing process might bring these natural frequencies even closer, leading to improper testing conditions. Consequently, a redesign would be required. Often, small changes in one of the dimensions (like the length or width) are enough to move the natural frequencies apart from each other. Figure 7 presents a process flowchart summarizing the specimen design procedure.

### 2.4. Experimental Procedure

The present section will provide details regarding the specimen preparation, experimental procedure, and measurement systems. Table 4 presents an overview of the equipment used during very-high-cycle fatigue tests. All tests were performed in a Shimadzu USF-2000 machine, which has its operation frequency in the range of 20 kHz ± 500 Hz. The specimens were tested using a full-reversible load ratio (R = −1). Due to its working principle, the machine can only work within a certain frequency range. Therefore, when crack nucleation and growth happen, the specimen-resisting cross-section will be reduced, and consequently, its stiffness and its natural frequency will be smaller than prior to crack propagation. Therefore, the machine will automatically stop after a certain crack length (due to the changes in the system natural frequency), and the test will stop before the specimen’s complete separation. Consequently, it is necessary to perform an additional separation step in order to allow the observation of the fracture surfaces.

The fatigue tests were organized into three main stress levels: 150, 140, and 130 MPa. Initially, three specimens were tested for each stress level; this repetition at a given stress level is used to ensure certain repeatability, since S-N curves are associated with certain levels of scatter/dispersion. Next, two additional tests will be performed for the stress level with the greatest scatter, and one specimen for the stress level with intermediate dispersion. Last, if no run-out is found, two tests will be performed for a lower stress amplitude (120 MPa for the present case).

During tests, displacements were measured using a vibrometer Polytec VibroOne (the velocity and the displacement were measured through numerical time integration), and a sampling rate of 1.25 MHz was used.

In order to validate the numerical model and the strain/stress computed, measurements of strain were performed and later compared with the numerical data; this was accomplished by using strain gauges. For the strain measurements, a sampling rate of 200 kHz was used. Additionally, the experimental and numerical natural frequencies were also compared.

**Figure 8:**
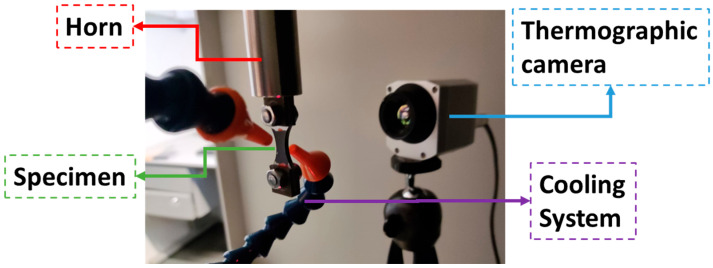
Cooling and temperature measurement systems.

Due to its high excitation frequency (approximately 20 kHz), ultrasonic fatigue tests can lead to high temperatures during tests, which may impact the fatigue response of a given material. Consequently, it is essential to monitor temperatures during tests. An Optris PI 400i infrared camera was used during tests, and the specimen’s testing section was painted black in order to ensure a known surface emissivity. Compressed air was also used to ensure adequate cooling of the specimen testing section (the hottest region of the specimen). Maximum temperatures superior to 40 °C were not allowed during tests in order to minimize the temperature effect on the fatigue behaviour. In order to achieve these temperatures, a combination of air cooling and intermittent excitation was used (a common practice in ultrasonic fatigue testing). An excitation period of 0.4 s was used (approximately 8000 cycles per pulse), and an equal resting time was applied. Figure 8 presents an overview of the temperature monitoring and cooling setup.

**Figure 9:**
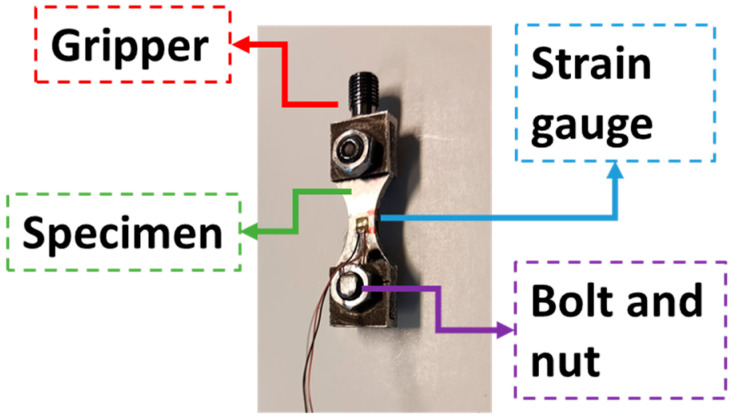
Griping system, specimen, and strain gauge.

Regarding the strain measurements, Figure 9 presents an overview of the gripping section, specimen, and a strain gauge. The strain gauge was attached to the middle length of the specimen, and at its centre. Although this is not the region of the maximum stress (and, consequently, the crack will not nucleate at this region), it is a region where the stress field does not present any significant variation (along the specimen length and width), while having high stress amplitudes.

**Figure 10:**
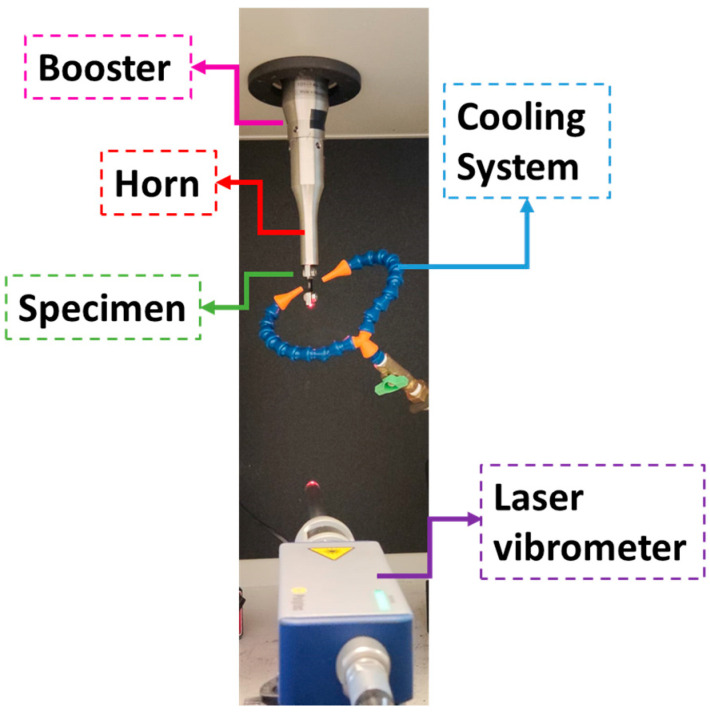
Displacement measurement system and testing apparatus.

Regarding the displacement measurements, Figure 10 presents the measurement setup. The displacement measurements were performed at the lowest portion of the bottom gripper, and they were performed along the specimen’s longitudinal direction. Due to symmetry (the specimen is excited in its 1st longitudinal model), the displacement at the horn and the uppermost portion of the top gripper (both are attached) will have the same magnitude as the measured one.

**Figure 11:**
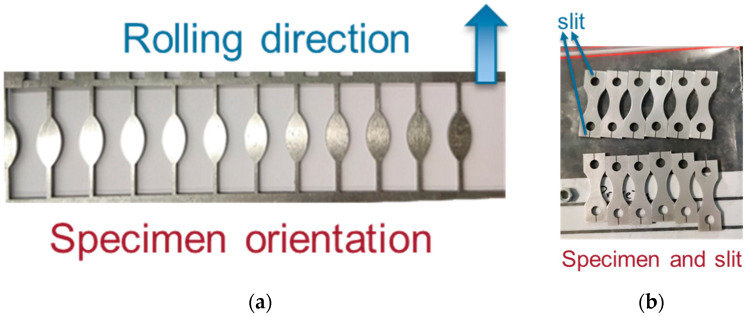
Specimen orientation and manufacturing: (**a**) Rolling and specimen orientation; and (**b**) specimen after WEDM.

As previously discussed, one of the main goals of the present work is to provide a comprehensive design procedure for testing specimens obtained from thin-walled components. The chosen material was an aluminium 6082, and this decision was based on the fact that this alloy is frequently applied in low-thickness components obtained via forming processes, like sheets and tubes. A 2 mm-thick sheet was used, and a Wire Electron Discharge Machine (WEDM) was employed to obtain the specimen’s geometry. Figure 11a presents a sheet after specimen manufacturing. The specimens were manufactured to ensure that their longitudinal direction (maximum stress orientation) was parallel to the rolling direction of the forming process.

Figure 11b presents the specimens after the WEDM process. A slit can be noticed between the specimens’ holes and their external contour (outline). This was performed in order to avoid any additional manufacturing steps, such as the need for a machining process, in order to manufacture the holes. It is important to emphasize that this slit will not have a significant impact on the mechanical resistance of the specimen at the gripping section. As previously discussed, the gripper and specimens are held together due to the friction force between them (due to the pre-load resulting in a normal force), and no significant forces are applied at the specimen hole region by the bolts (through hole).

After WEDM, all specimens were sanded and polished at the testing section. All four faces of the testing section were polished (two side ones and two front ones), and an average roughness (Ra) of approximately 0.10 μm was obtained. The gripping region of the specimen was not polished in order to provide a high friction coefficient between it and the gripper. In total, 14 specimens were manufactured and tested. Additionally, Scanning Electron Microscopy (SEM) was used to analyze the fracture surfaces.

Two grippers were manufactured by WEDM as well. As discussed in Section 2.1, in order to minimize the overall mass of the gripping system, titanium was used as the gripper material (Ti4Al6V), having approximately half the mass of steel grippers (using the same geometry). Additionally, due to its lower elasticity modulus when compared with steel, the titanium grippers have lower stiffness when subjected to the bending moments due to the bolt pre-load, assuring a higher contact force between the specimen and the gripper (and, consequently, higher friction forces). Regarding the top gripper, an external thread was machined with the purpose of attaching the specimen and gripping system to the horn of the machine (Figure 3).

## 3. Results and Discussion

This section will present the obtained experimental and numerical results and a brief discussion and comparison between them. Firstly, the obtained specimen geometry will be discussed, followed by the validation of the numerical model through experimental measurements. Lastly, the experimental fatigue data and fracture surfaces will be presented.

### 3.1. Base Alloy Characterization

The material investigated in this study is a rolled EN AW-6082 aluminium alloy sheet (EN AW-Al Si1MgMn) delivered in a peak-aged condition, nominally equivalent to a T6/T5x temper. The nominal chemical composition is within the specification range of EN 573-3:2019 for EN AW-6082 (Table 1), with Si and Mg as the main alloying elements and Mn additions for grain-structure and dispersoid control.

**Figure 12:**
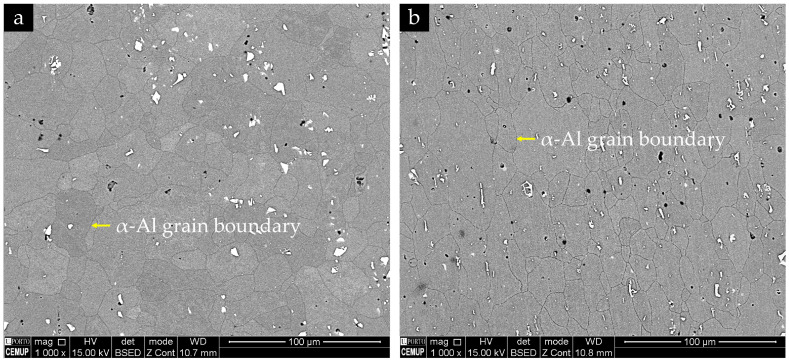
BSE images of the EN AW-6082 base alloy showing the equiaxed recrystallised α-Al grain structure: (**a**) top surface of the fatigue specimen in the fatigue test clamping area; and (**b**) longitudinal section in the same region. Coarse bright intermetallic particles and dark pores/pulled-out features are observed within the α-Al matrix.

The microstructure of the base alloy exhibits an almost equiaxed, recrystallised α-Al grain structure, with grain sizes of the order of a few tens of micrometres, as shown in Figure 12. No pronounced grain elongation is observed, despite the rolled condition of the sheet, indicating an essentially recrystallised matrix. In contrast, the distribution of second-phase particles and defects is clearly heterogeneous: coarse bright particles and dark rounded features are observed both on the top surface and in longitudinal sections.

**Figure 13:**
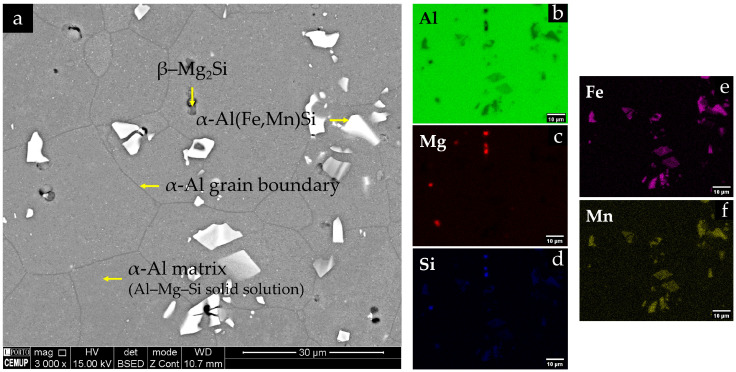
BSE image of the EN AW-6082 base alloy: (**a**) α-Al matrix, grain boundaries, and coarse second-phase particles on the top surface of the fatigue specimen (clamping area), showing bright angular particles that correspond to α-Al(Fe,Mn)Si intermetallics, with grey particles consistent with β-Mg_2_Si; (**b**–**f**) corresponding EDS maps for Al (**b**), Mg (**c**), Si (**d**), Fe (**e**), and Mn (**f**).

BSE imaging combined with EDS analyses, shown in Figure 13, indicates that the bright angular intermetallic particles are enriched in Fe and Mn and correspond to α-Al(Fe,Mn)Si-type phases, with compositions close to Al_12_(Fe,Mn)_3_Si in agreement with reported α-AlFe(Mn)Si phases in AA6082 during homogenization [33]. Grey particles enriched in Mg and Si but depleted in Fe are consistent with coarse Mg_2_Si-type phases. Numerous submicron bright spots inside the α-Al grains are interpreted as fine Fe/Mn-containing dispersoids, as depicted in Figure 13. Dark, rounded features correspond to a combination of fine porosity and pulled-out particles. In the longitudinal section, the coarse α-Al(Fe,Mn)Si particles and pores tend to form preferentially aligned strings along the rolling direction, revealing a microstructural banding of intermetallics and defects, even though the α-Al grains themselves remain nearly equiaxed, as observed in Figure 12b.

**Figure 14:**
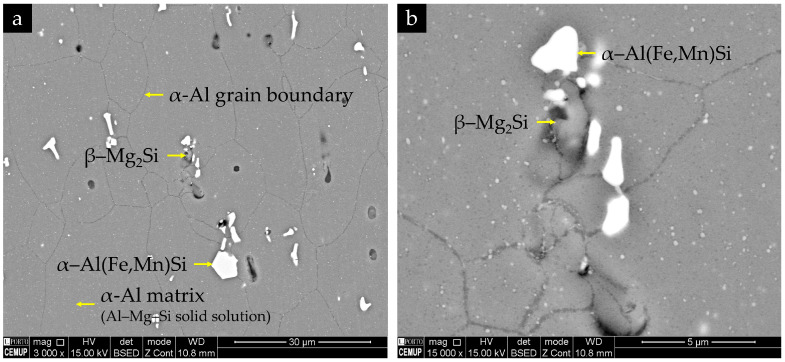
BSE images of the EN AW-6082 base alloy in longitudinal section, taken in the clamping area of the fatigue specimen: (**a**) overview at 3000× showing the equiaxed α-Al matrix (Al–Mg–Si solid solution), α-Al grain boundaries, and the heterogeneous distribution of coarse second-phase particles; and (**b**) higher magnification image at 15,000× highlighting clusters of bright α-Al(Fe,Mn)Si intermetallics, grey β-Mg_2_Si particles, and submicron fine Fe/Mn-containing dispersoids inside the α-Al grains.

The hardness of the base material is 111 ± 4 HV1 (7 measurements), measured in regions of the clamping area unaffected by fatigue damage. This value lies within the typical range reported for peak-aged AA6082 and is consistent with the observed microstructure: an equiaxed α-Al matrix containing a dispersion of coarse α-Al(Fe,Mn)Si and Mg_2_Si-type particles together with fine Fe/Mn-rich dispersoids (see Figure 14), as well as nanoscale Mg–Si-strengthening precipitates (β″/β′) that govern the peak-aged temper but remain below the resolution of SEM. This value lies within the typical range reported for a peak-aged AA6082-T6 sheet [34].

### 3.2. Specimen Geometry

As previously discussed, the design process was iterative, mainly due to factors such as the following: achieving a desired natural frequency; reaching a relation between the minimum and maximum displacement that the machine is capable of, and its relation with the stress amplitudes at the testing section; relation between longitudinal separation forces and friction between the specimen and gripper; and the presence of additional (non-desired) modes with corresponding frequencies near the desired mode (1st longitudinal vibration mode of the specimen).

**Figure 15:**
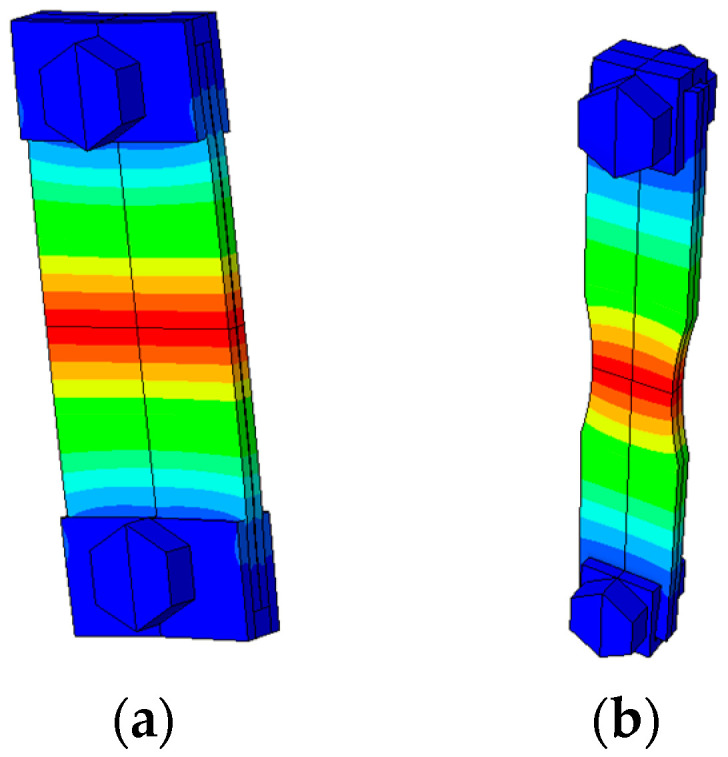
Example of preliminary geometries (displaying longitudinal axial stress magnitude field): (**a**) Straight specimen; and (**b**) specimen with large testing section width.

Figure 15 presents examples of previously analyzed geometries and their respective longitudinal normal stress fields (maximum at the specimen centre for the desired vibration mode). Regarding Figure 15a, a very simple straight specimen geometry was used. However, due to its relatively large cross-sectional area at the testing section, a low specimen gain was obtained. This means that the machine is not able to test the specimen at the desired stress amplitudes, resulting in tests with possibly no crack nucleation (due to insufficient stress amplitude). Figure 15b presents an intermediate specimen design. This time, the cross-section area was reduced by reducing the testing section width. This modification would result in a higher specimen gain, meaning that now the machine was able to achieve the desirable stress levels. However, the testing section was still relatively large, meaning that larger inertial forces would be required to achieve higher stress amplitudes. As previously discussed, the inertial forces are dominated by the outer regions of the specimen, regions where the gripper system is located. Consequently, the obtained solutions presented a large longitudinal separation force between the gripper and specimen, meaning that the obtained friction was not large enough to maintain the specimen and gripper with no relative motion. Therefore, additional changes in the specimen design (focused this time on reducing the cross-section area of the testing section) must be performed.

**Figure 16:**
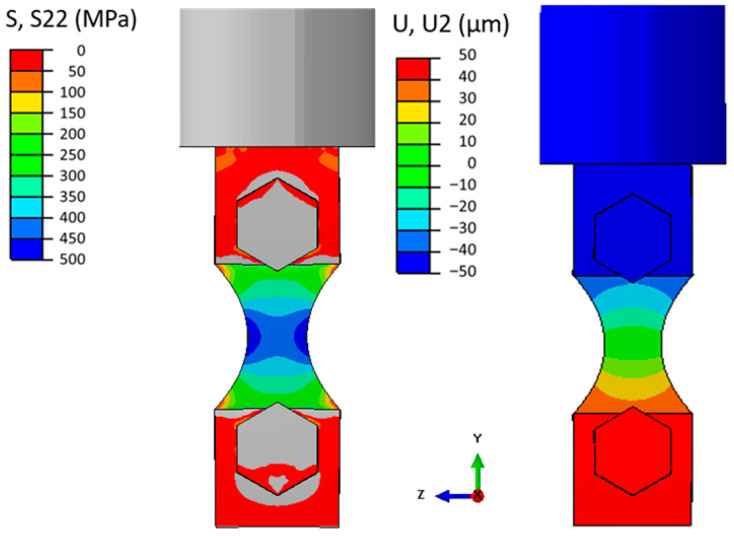
Longitudinal displacement and normal stress distribution.

Regarding the final design, additional reductions at the testing sections were performed. Figure 16 presents the final geometry, where the longitudinal stress and displacement fields can be seen. The presented fields are relative to the maximum machine capacity, resulting in a maximum displacement amplitude of approximately 55 μm at the machine horn. Due to the symmetries in the 1st longitudinal mode, this displacement magnitude is the same as the one found at the opposite side of the specimen (bottom gripper). Additionally, being the 1st longitudinal vibration mode, maximum stress levels were obtained at the central section of the specimen, and these stress amplitudes are minimal at the ends of the piece (top and bottom gripper regions).

**Figure 17:**
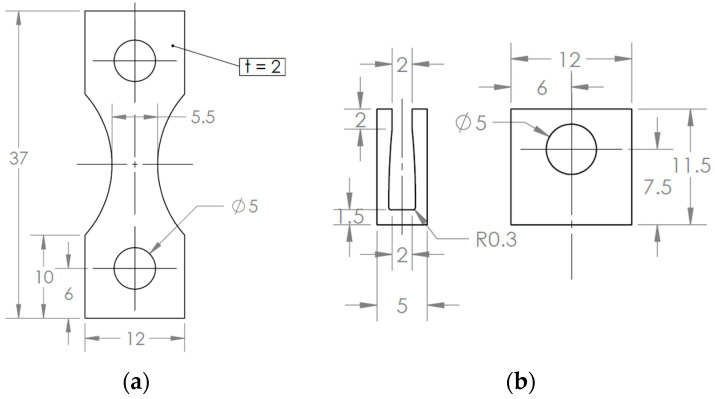
Specimen and gripper geometry, main dimensions in mm: (**a**) Specimen drawing; and (**b**) bottom gripper drawing.

For this design, a longitudinal natural frequency of 19.94 kHz was obtained. Table 5 presents a summary of the obtained properties. Due to the specimen radius (and mass distribution), a stress concentration was obtained at the edges of the testing section (middle length of the specimen). A 1.09 stress concentration factor was presented (when comparing maximum and nominal stress), and a gain of 9.3 MPa/µm was computed. Moreover, at the central portion of the testing section, a gain of 7.7 MPa/µm was obtained. This value will be relevant later, when the measured experimental strain (at this central middle length position) will be used as a comparison with the strain and stress levels obtained numerically. The specimen and bottom gripper main dimensions are presented in Figure 17.

**Figure 18:**
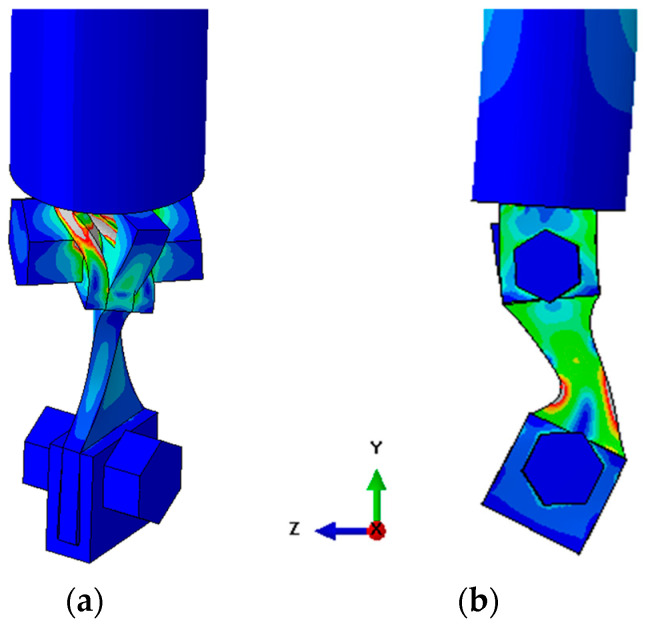
Specimen displacement and stress distribution: (**a**) Torsion mode, 17.88 kHz; and (**b**) bending moment in x-direction, 20.08 kHz.

Lastly, it is essential to verify if there are other vibration modes close to the mode of interest. In the previous analyses, it was possible to utilize symmetry (like 1/4th or 1/8th) once the mode of interest presented symmetry along the three principal orthogonal planes. However, a full model (no symmetry) must be used to obtain all adjacent vibration modes. Figure 18a presents the adjacent vibration mode, under the mode of interest, and the von Mises equivalent stress filed is presented. It can be seen that for the obtained mode, the maximum stresses are not located at the testing section of the specimen (central portion). For this case, a torsion mode of 17.88 kHz was obtained, and the frequency difference was very large (outside the operating range of the machine, and thus it does not hinder the functionality of the system). Regarding the other adjacent mode, Figure 18b presents the vibration mode above the mode of reference, and the von Mises equivalent stress field is also presented. For this case, the frequency differences are smaller (approximately 104 Hz), but within an acceptable range. Small changes could be applied to some specimen dimensions (like its length or testing section) in order to determine if greater frequency differences between the modes could be achieved. It is important to emphasize that, due to its small stiffness in some direction (like in the translational x-direction or rotational z-direction), several other modes are present, potentially leading to difficulties in finding large frequency differences between modes.

### 3.3. Numerical Model Validation

**Figure 19:**
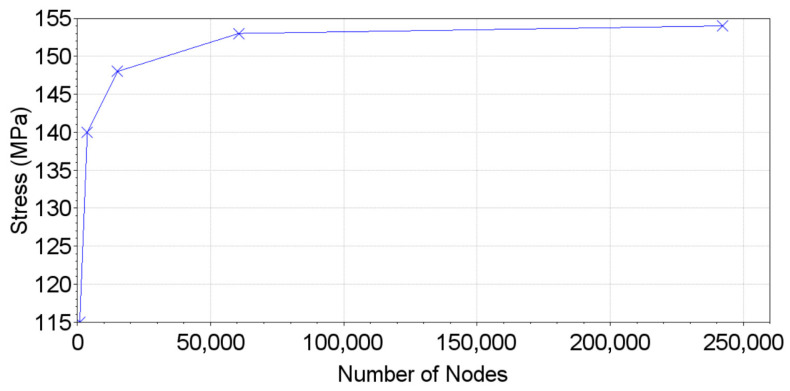
Mesh convergence analysis.

The present section will provide a comparison between the measured experimental data (reference) and the numerical data. A mesh convergence analysis was performed with the full simulation model, and the maximum normal stress amplitudes (along the longitudinal direction) for the desired vibration mode (longitudinal) were computed at the testing section, and these are presented in Figure 19. A maximum variation of 2% was chosen as the criterion for mesh convergence. The model used consisted of approximately 60,000 nodes.

**Figure 20:**
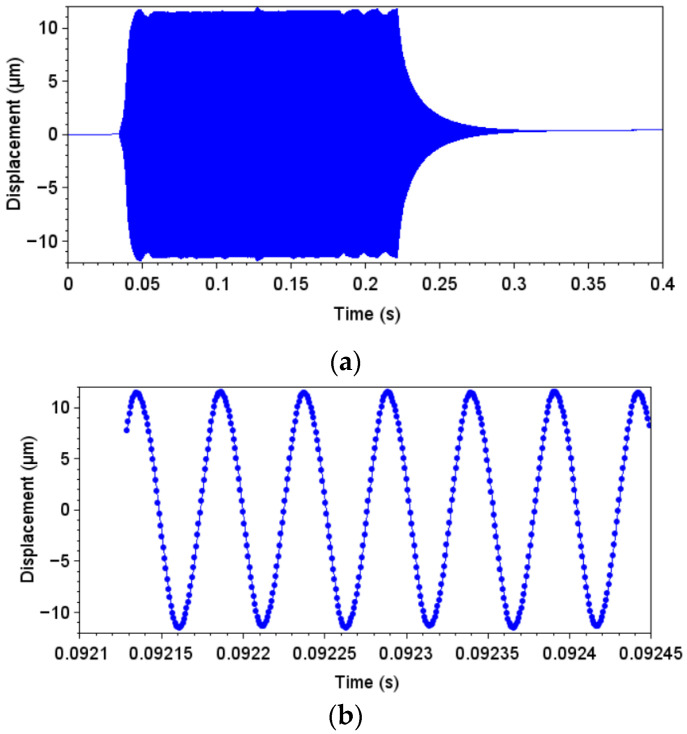
Displacement measurements: (**a**) Displacement, overview; and (**b**) displacement, zoomed-in view.

First, the displacement was measured using the laser vibrometer at the bottom of the specimen. A loading condition of approximately 20% of the maximum machine displacement was used for the measurements. Figure 20a presents an overview of the measured displacement during an excitation pulse, while Figure 20b presents a zoomed-in view of a few vibration cycles. An average displacement amplitude of 11.4 μm was measured.

**Figure 21:**
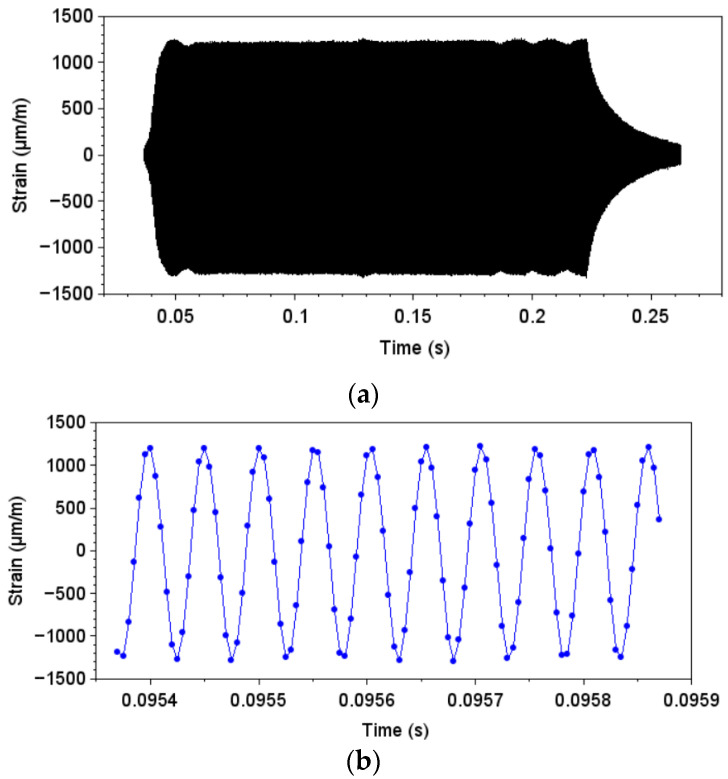
Strain measurements: (**a**) Strain, overview; and (**b**) strain, zoomed-in view.

In addition to the displacement measurements, the strain was obtained during the same excitation pulse. As previously discussed, the strain was measured at the central portion of the specimen, in the middle length of the specimen. At this region, the relation between stress and displacement computed was 7.7 MPa/μm (also referred to as gain). It is important to emphasize that this region is not the one corresponding to the maximum stress amplitudes. The region of maximum stress amplitudes is also at the middle length of the specimen, but at the edge region (and the gain is 9.2 MPa/μm). Using the experimental displacement measurement and the relation between stress and displacement (gain) obtained numerically, it is possible to estimate the stress levels. For the present case, stress obtained by this approach would be 87.3 MPa (at the specimen region). Figure 21 presents the strain measurements, where Figure 21a presents the total excitation pulse measurement, while Figure 21b shows a zoomed-in view of a few loading cycles. An average strain amplitude of 1254 μm/m was measured. When computing the stress amplitude by using the elasticity modulus (70 GPa), a stress amplitude of 87.8 MPa was obtained. The comparison between the stress computed based on the numerical approach (experimental measurements of displacement and stress/displacement relation obtained numerically) and the experimental approach (strain gauge measurements) presented a difference of approximately 0.5%. Therefore, it can be stated that the numerical model was able to properly capture the relation between displacement and strain.

Next, the experimental and numerical frequencies were compared. An experimental frequency of 19.74 Hz was obtained, while the numerical frequency computed was 19.94 Hz, resulting in a difference of approximately 1.0%. Therefore, the numerical model was able to properly compute the natural frequency of the system (machine, grippers, and specimen assembly).

### 3.4. Fatigue Tests and S-N Curve

**Figure 22:**
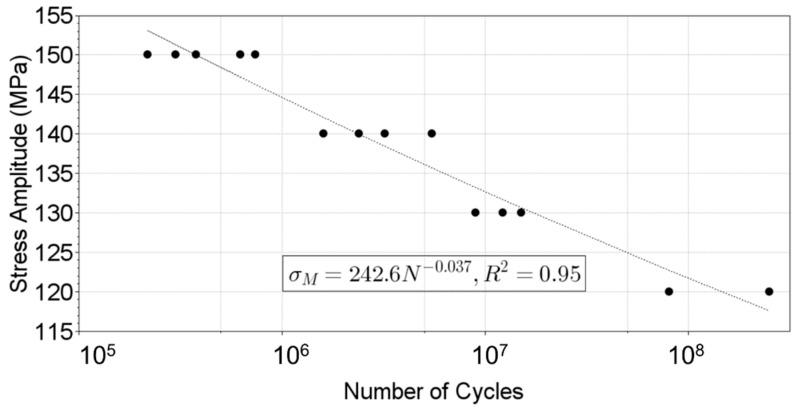
S-N curve for the 6082-sheet material.

As previously discussed, 14 specimens were manufactured and tested. Four stress amplitudes were analyzed: 120, 130, 140, and 150 MPa. Figure 22 presents a summary of the obtained S-N curve. The stress amplitudes correspond to the maximum amplitudes present in the specimen, at the middle length of it, and at the edges (Figure 17). Relative low variance was obtained for the data (R^2^ = 0.94), meaning that a homogeneous material and surface finishing were present. No run-outs were obtained in the present experimental analysis, even when small stress amplitudes were present (140 MPa, for example, approximately 45% of the material UTS), and failures were observed (at around a million cycles for the present example).

**Figure 23:**
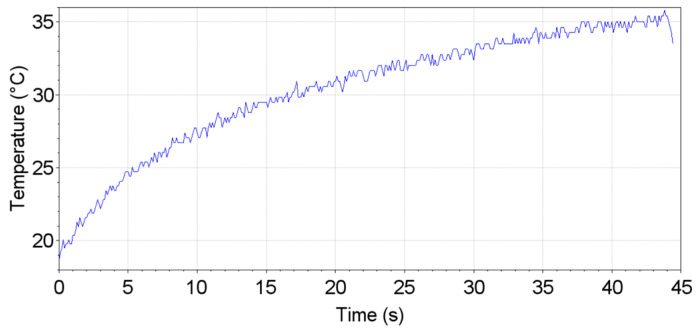
Temperature measurement during VHCF test.

Regarding the temperature control during tests, Figure 23 presents a temperature measurement during one of the fatigue tests, where the highest stress amplitudes were applied (150 MPa). As discussed, the temperatures were measured at the testing section, and the maximum temperature at this region is presented. With maximum temperatures around 35 °C, it can be assured that their influence on S-N curves can be neglected, and the obtained results were very similar to room temperature data.

### 3.5. Literature Comparison

**Figure 24:**
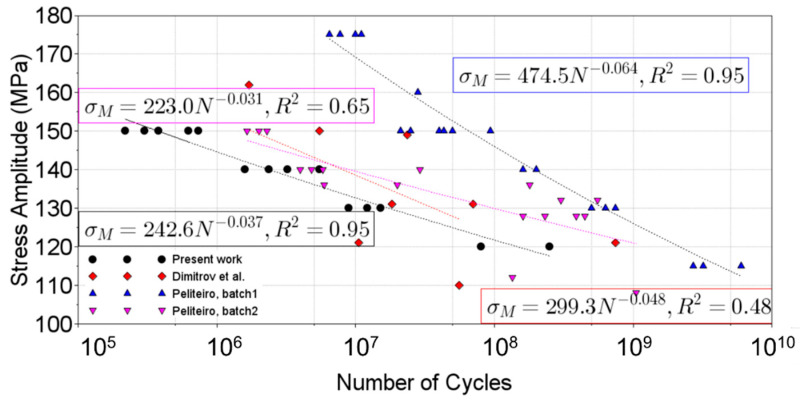
VHCF results and literature comparison [29,30].

In order to validate the proposed methodology and results obtained, the present section will encompass a brief comparison with the literature results. Figure 24 presents the S-N curve for the present work (in black) and the results obtained from Dimitrov et al. [29] and Peliteiro [30], which characterizes the VHCF behaviour of 6082 for hourglass specimens (while the present analysis was performed for flat ones). It can be observed that the obtained S-N curves are very similar to the presented ones, except for the 2nd batch of Peliteiro’s work. In that work, the authors attributed the discrepancy between batches as being due to different material supplied conditions, like cold working. This is clear in Peliteiro’s work, where two different batches resulted in significant differences. Therefore, it can be stated that the proposed specimen design and testing procedure lead to results comparable with the literature available in VHCF. However, it is important to emphasize that this comparison’s main goal is just to provide an overall view of the present geometry, and the testing campaigns compared were generated for different specimens’ geometries and material conditions.

### 3.6. Fracture Surfaces

**Figure 25:**
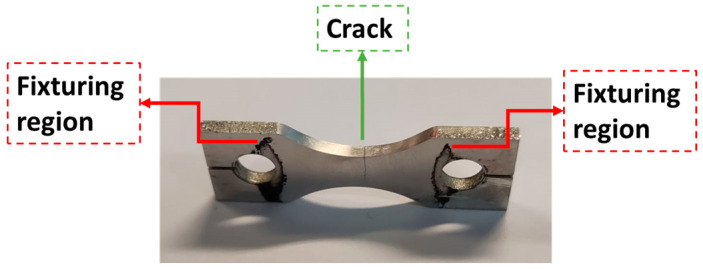
Overview of specimen and crack.

The present section will provide a brief discussion regarding the specimen fracture surfaces. Figure 25 presents an example of a specimen after the fatigue tests, where the crack can be seen at the central section (region comprising the highest stress amplitudes). As previously discussed, the machine loading stops once it detects defined variations in the system’s natural frequencies. Therefore, the tests stop before the complete specimen separation, and the mechanical separation was performed after.

**Figure 26:**
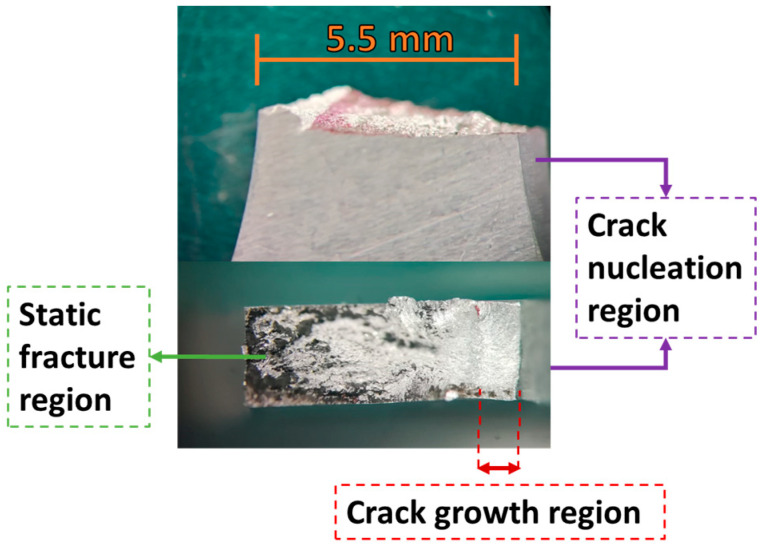
Macroscopic view of fracture surface.

Figure 26 presents an overview of a specimen fracture surface, from both a front and a top view. The regions of fatigue fracture (right) and static fracture (left, performed after the VHCF tests) can be seen. An uneven surface can be seen at the static fracture region, and due to the peaks and valleys at the fracture surface, some regions appear out of focus (different focal distances).

**Figure 27:**
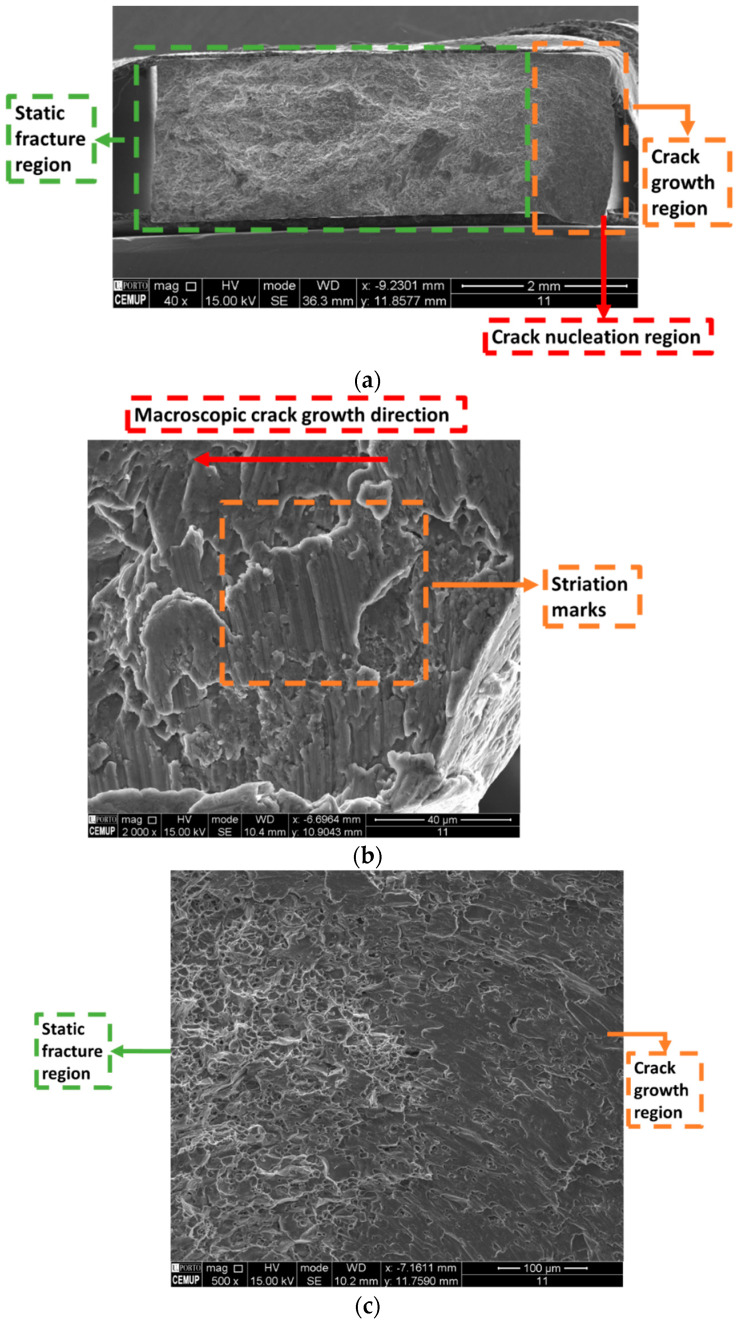

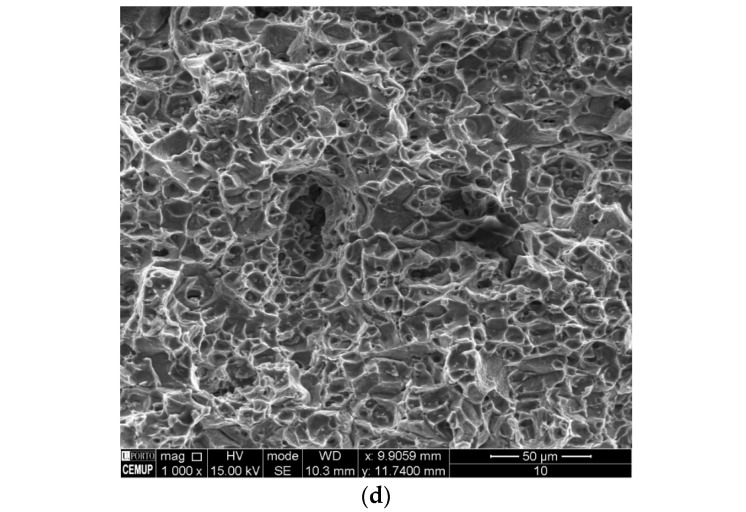
Specimen fracture surface: (**a**) Fracture surface, macroscopic view; (**b**) striation marks; (**c**) transition region, fatigue (right) and static fracture (left); and (**d**) static fracture region.

The SEM images of a specimen fracture surface can be seen in Figure 27. A macroscopic view of the fracture surface can be seen in Figure 27a, where the crack nucleation region can be seen at the rightmost portion of the image. Striation marks can be seen near the crack nucleation region, as shown in Figure 27b, indicating that the material tested presented good ductility. Moreover, Figure 27c presents the transition region between fatigue crack growth (right) and static fracture (left). Figure 27d presents an image of the static fracture region, where dimples can be seen, characterizing a ductile fracture.

No major differences were observed between the different fracture surfaces for different stress levels, once a relatively small variation in stress amplitudes was applied for different tests (being 150 MPa the highest, while 120 MPa was the smallest). A similar behaviour was obtained for crack nucleation, where it happened at the shorter edges of the testing section (as shown in Figure 27a), the rightmost region.

**Figure 28:**
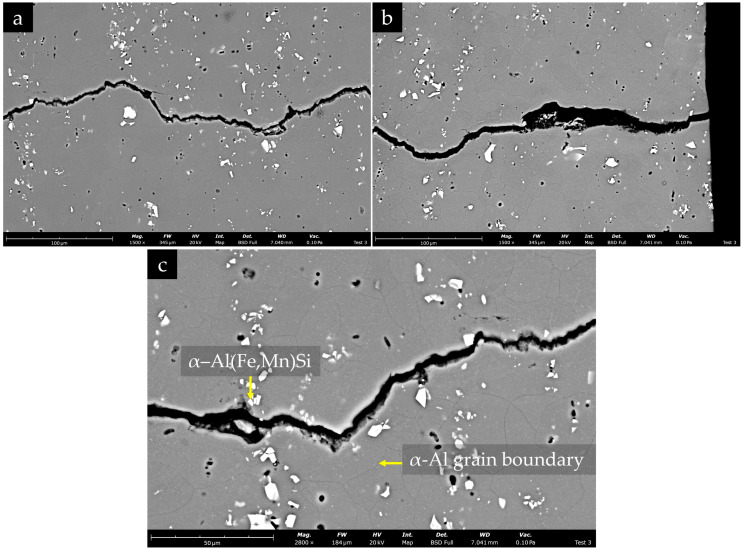
BSE images of the fatigue crack path in the EN AW-6082 base material: (**a**,**b**) overview of the crack propagating through the α-Al matrix, with a complex path crossing grains and occasionally deflecting at grain boundaries; (**c**) higher-magnification image showing the interaction between the crack and a coarse α-Al(Fe,Mn)Si particle located near an α-Al grain boundary. Dark, rounded features correspond to fine porosity and/or pulled-out cavities.

As shown in Figure 28, the fatigue crack follows a complex path through the α-Al matrix, and it is predominantly transgranular but has local deflections at grain boundaries. The crack frequently interacts with coarse α-Al(Fe,Mn)Si particles (see Figure 28c) and nearby pores, which act as local stress raisers and promote crack advance. Some particles are cracked or debonded along the crack edges.

## 4. Conclusions and Future Work

The design methodology proposed was shown to be capable of achieving satisfactory specimen geometry, encompassing several key factors, such as the following: specimen frequency, stress distribution, and the relation between displacement and stress. Moreover, the obtained numerical data, such as strain amplitudes and the natural frequency, were validated through experimental measurements (displacement and strain gauge data). Lastly, the obtained specimen geometry was used to characterize the material in the VHCF regime, obtaining an S-N curve. Fatigue crack initiation and growth in EN AW-6082 are thus governed primarily by coarse intermetallic particles and pores embedded in an otherwise recrystallised α-Al matrix. Additionally, similar crack nucleation and propagation were observed in all fracture surfaces, despite the different stress amplitudes applied during fatigue tests.

Regarding future works, it would be interesting to compare the obtained results with traditional fatigue tests (lower excitation frequencies) in order to understand the frequency effect in fatigue properties of this material, 6082. Moreover, testing different geometries, for example, larger specimens (for example, different length and width, with the same component thickness), would provide an interesting comparison regarding the size effect in fatigue properties. In order to provide more robust validation of the proposed design methodology, testing different materials (using the same design workflow) would provide valuable information regarding the proposed method. Lastly, the same design methodology could be used to design specimens for Fatigue Crack-Growth Rate, helping understand how cracks propagate for very small stress-intensity factors.

## Figures and Tables

**Table 1:** Nominal chemical composition of the EN AW-6082 aluminium alloy, adapted from [31] (wt.%).

Alloy Designation		Si	Fe	Cu	Mn	Mg	Cr	Zn	Ti	Others		Al
Numerical	Chemical symbols	0.70–1.30	≤0.50	≤0.10	0.40–1.00	0.60–1.20	≤0.25	≤0.20	≤0.10	Each	Total	Bal.
EN AW-6082	EN AW-Al Si1MgMn									≤0.05	≤0.15	

**Table 2:** Effect of the increases for main parameters in specimen’s properties.

Parameter	Stiffness, K	Mass, M	Natural $\mathrm{Frequency},\;\omega_{n}$	Notes
Specimen young modulus, E	↑	-	↑	
Specimen density, ρ	-	↑	↓	
Specimen length, L	↓	↑	↓	
Specimen testing section width, W_i_	↓	↓	↓	
Specimen gripping section width, W_o_	-	↑	↓	Influence bolstered if gripping system increases in size
Specimen thickness, t	↑	↑	↓	Influence heavily dependent on gripping system size
Gripping section dimensions	-	↑	↓	
Gripping section density	-	↑	↓	

**Table 3:** Main properties of ultrasonic machine and specimen properties.

Type	Parameter	Information
Machine	Operating frequency (Hz)	20,000 ± 500
	Minimum displacement amplitude (µm)	11
	Maximum displacement amplitude (µm)	55
Specimen	Material	6082 Aluminium Alloy
	Elasticity Modulus (GPa)	70
	Density (kg/m^3^)	2700
	Ultimate tensile strength (MPa)	310

**Table 4:** Summary of experimental apparatus.

Equipment or Sensor	Name
Ultrasonic fatigue machine	Shimadzu USF-2000 (Tokyo, Japan)
Displacement sensor	Polytec VibroOne (Waldbronn, Germany)
Strain gauge	Micro-Measurements MMF402183 (Malvern, PA, USA)
Infrared camera	Optris PI 400i (Berlin, Germany)
Surface roughness measurement	Mitutoyo SJ-210 (Kawasaki, Japan)

**Table 5:** Main dimensions and stress parameters of specimen’s final geometry.

Parameter	Value
Length	37 mm
Testing section width	5.5 mm
Gripping section width	12 mm
Thickness	2 mm
Maximum gain	9.2 MPa/µm
Minimum gain	7.7 MPa/µm
Maximum stress	506 MPa

## Data Availability

The data presented in this study are available on request from the corresponding author due to restrictions associated with project/funding data.

## Abbreviations

The following abbreviations are used in this manuscript:

VHCF	Very-high-cycle fatigue
WEDM	Wire Electron Discharge Machine
SEM	Scanning Electron Microscopy
